# Supplementary material for: Low-Level Tolerance to Fluoroquinolone Antibiotic Ciprofloxacin in QAC-Adapted Subpopulations of Listeria monocytogenes
Source: Microorganisms. 2021 May 13;9(5):1052. doi: 10.3390/microorganisms9051052 (PMC8153124; doi:10.3390/microorganisms9051052)
Supplement: Supplementary file 1 [file microorganisms-09-01052-s001.zip › microorganisms-1216704-supplementary.pptx]

## Slide 1
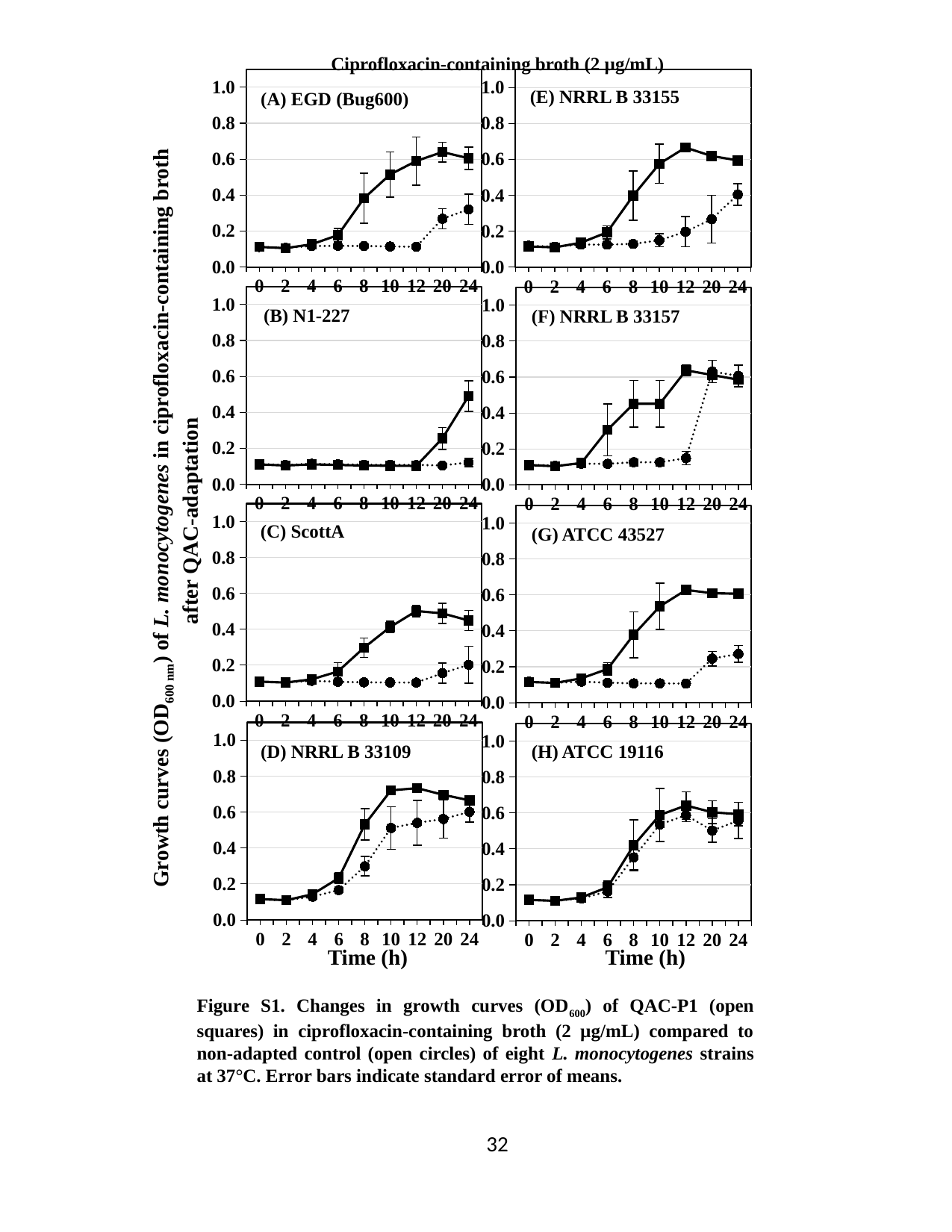

Ciprofloxacin-containing broth (2 µg/mL)
### Chart
| Category | 0S | p1 |
|---|---|---|
| 0 | 0.11099999999999999 | 0.11133333333333333 |
| 2 | 0.1065 | 0.10433333333333333 |
| 4 | 0.11649999999999999 | 0.12566666666666668 |
| 6 | 0.1175 | 0.17683333333333331 |
| 8 | 0.11666666666666665 | 0.38200000000000006 |
| 10 | 0.11366666666666665 | 0.5143333333333333 |
| 12 | 0.11199999999999999 | 0.5898333333333333 |
| 20 | 0.26808333333333334 | 0.6395 |
| 24 | 0.32 | 0.6048333333333333 |
### Chart
| Category | 0S | p1 |
|---|---|---|
| 0 | 0.119 | 0.11499999999999999 |
| 2 | 0.11366666666666665 | 0.10983333333333334 |
| 4 | 0.12516666666666668 | 0.13566666666666663 |
| 6 | 0.12516666666666665 | 0.19316666666666668 |
| 8 | 0.12916666666666665 | 0.3973333333333333 |
| 10 | 0.14916666666666667 | 0.5746666666666665 |
| 12 | 0.19666666666666666 | 0.6655 |
| 20 | 0.2668333333333333 | 0.6185 |
| 24 | 0.4038333333333333 | 0.5941666666666666 |(E) NRRL B 33155
(A) EGD (Bug600)
Growth curves (OD600 nm) of L. monocytogenes in ciprofloxacin-containing broth after QAC-adaptation
### Chart
| Category | 0S | p1 |
|---|---|---|
| 0 | 0.11249999999999999 | 0.11033333333333332 |
| 2 | 0.10716666666666665 | 0.104 |
| 4 | 0.11383333333333333 | 0.11049999999999999 |
| 6 | 0.11083333333333333 | 0.107 |
| 8 | 0.10849999999999999 | 0.10400000000000002 |
| 10 | 0.10799999999999998 | 0.10266666666666668 |
| 12 | 0.10733333333333334 | 0.10183333333333333 |
| 20 | 0.1045 | 0.2555 |
| 24 | 0.12133333333333333 | 0.4911666666666667 |
### Chart
| Category | 0S | p1 |
|---|---|---|
| 0 | 0.11233333333333333 | 0.11 |
| 2 | 0.10666666666666667 | 0.1035 |
| 4 | 0.11783333333333333 | 0.12183333333333334 |
| 6 | 0.117 | 0.30633333333333335 |
| 8 | 0.12583333333333335 | 0.451 |
| 10 | 0.12583333333333335 | 0.451 |
| 12 | 0.14866666666666664 | 0.6373333333333333 |
| 20 | 0.6306666666666666 | 0.6114999999999999 |
| 24 | 0.6065 | 0.5851666666666667 |(B) N1-227
(F) NRRL B 33157
### Chart
| Category | 0S | p1 |
|---|---|---|
| 0 | 0.10933333333333334 | 0.10883333333333334 |
| 2 | 0.10416666666666667 | 0.10333333333333335 |
| 4 | 0.11316666666666668 | 0.12133333333333333 |
| 6 | 0.10799999999999998 | 0.165 |
| 8 | 0.10483333333333333 | 0.29716666666666663 |
| 10 | 0.104 | 0.4121666666666666 |
| 12 | 0.1035 | 0.5018333333333334 |
| 20 | 0.1556666666666667 | 0.48899999999999993 |
| 24 | 0.20266666666666666 | 0.449 |
### Chart
| Category | 0S | p1 |
|---|---|---|
| 0 | 0.11699999999999999 | 0.1155 |
| 2 | 0.111 | 0.11 |
| 4 | 0.11716666666666666 | 0.13566666666666669 |
| 6 | 0.11116666666666668 | 0.18783333333333332 |
| 8 | 0.10766666666666667 | 0.37866666666666665 |
| 10 | 0.10766666666666667 | 0.5363333333333333 |
| 12 | 0.1065 | 0.6276666666666667 |
| 20 | 0.24550000000000002 | 0.6098333333333333 |
| 24 | 0.27133333333333337 | 0.6065 |(C) ScottA
(G) ATCC 43527
### Chart
| Category | 0S | p1 |
|---|---|---|
| 0 | 0.114 | 0.11616666666666665 |
| 2 | 0.1095 | 0.11016666666666668 |
| 4 | 0.12966666666666668 | 0.14316666666666666 |
| 6 | 0.16633333333333333 | 0.233 |
| 8 | 0.29866666666666664 | 0.5323333333333333 |
| 10 | 0.5121666666666668 | 0.7214999999999999 |
| 12 | 0.5406666666666666 | 0.7336666666666667 |
| 20 | 0.5619999999999999 | 0.6965 |
| 24 | 0.602 | 0.666 |
### Chart
| Category | 0S | p1 |
|---|---|---|
| 0 | 0.11599999999999999 | 0.11583333333333333 |
| 2 | 0.111 | 0.11083333333333334 |
| 4 | 0.12416666666666666 | 0.13 |
| 6 | 0.16316666666666665 | 0.18666666666666668 |
| 8 | 0.35300000000000004 | 0.41966666666666663 |
| 10 | 0.5356666666666667 | 0.589 |
| 12 | 0.5876666666666667 | 0.6416666666666667 |
| 20 | 0.5021666666666667 | 0.6040000000000001 |
| 24 | 0.5576666666666666 | 0.5935 |(D) NRRL B 33109
(H) ATCC 19116
Time (h)
Time (h)
Figure S1. Changes in growth curves (OD600) of QAC-P1 (open squares) in ciprofloxacin-containing broth (2 µg/mL) compared to non-adapted control (open circles) of eight L. monocytogenes strains at 37°C. Error bars indicate standard error of means.
32

## Slide 2
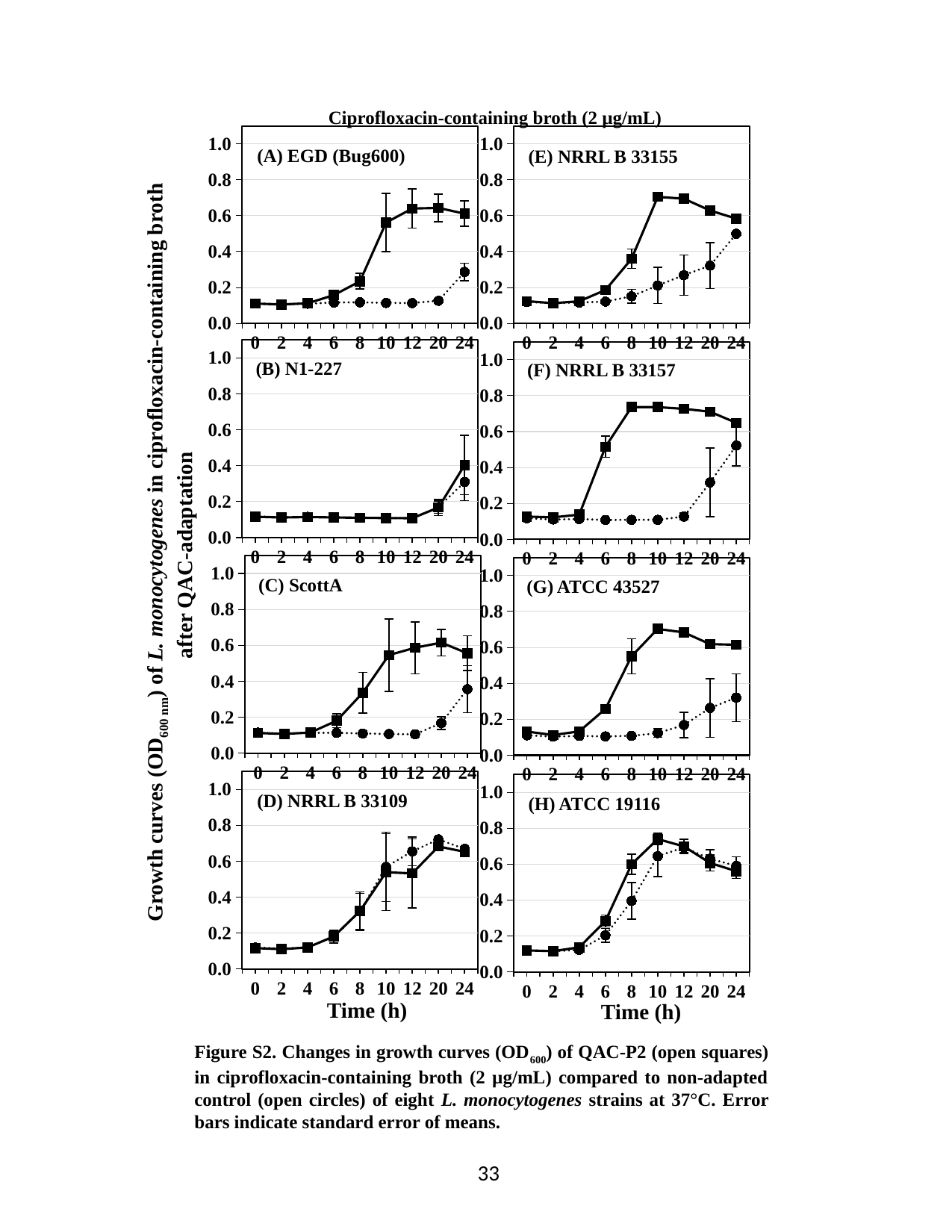

Ciprofloxacin-containing broth (2 µg/mL)
### Chart
| Category | 0S | p1 |
|---|---|---|
| 0 | 0.111 | 0.1105 |
| 2 | 0.106 | 0.10566666666666667 |
| 4 | 0.10983333333333334 | 0.11233333333333334 |
| 6 | 0.11633333333333333 | 0.15766666666666665 |
| 8 | 0.11783333333333333 | 0.23450000000000001 |
| 10 | 0.11399999999999999 | 0.5616666666666666 |
| 12 | 0.11366666666666665 | 0.6398333333333334 |
| 20 | 0.12616666666666668 | 0.6431666666666667 |
| 24 | 0.2866666666666667 | 0.6111666666666666 |
### Chart
| Category | 0S | p1 |
|---|---|---|
| 0 | 0.11966666666666666 | 0.124 |
| 2 | 0.1125 | 0.11266666666666665 |
| 4 | 0.11633333333333333 | 0.12366666666666666 |
| 6 | 0.12150000000000001 | 0.18600000000000003 |
| 8 | 0.15133333333333332 | 0.35966666666666663 |
| 10 | 0.21133333333333335 | 0.7045 |
| 12 | 0.2688333333333333 | 0.6945 |
| 20 | 0.32183333333333336 | 0.6288333333333334 |
| 24 | 0.49899999999999994 | 0.5835 |(A) EGD (Bug600)
(E) NRRL B 33155
Growth curves (OD600 nm) of L. monocytogenes in ciprofloxacin-containing broth after QAC-adaptation
### Chart
| Category | 0S | p1 |
|---|---|---|
| 0 | 0.11883333333333333 | 0.11599999999999999 |
| 2 | 0.11316666666666668 | 0.11166666666666665 |
| 4 | 0.11533333333333333 | 0.11433333333333333 |
| 6 | 0.1125 | 0.11233333333333334 |
| 8 | 0.1105 | 0.11016666666666668 |
| 10 | 0.10883333333333334 | 0.10883333333333334 |
| 12 | 0.10783333333333334 | 0.10783333333333334 |
| 20 | 0.1695 | 0.16716666666666666 |
| 24 | 0.30983333333333335 | 0.4038333333333333 |
### Chart
| Category | 0S | p1 |
|---|---|---|
| 0 | 0.11716666666666666 | 0.12666666666666665 |
| 2 | 0.11033333333333334 | 0.12233333333333334 |
| 4 | 0.11316666666666668 | 0.1375 |
| 6 | 0.10783333333333334 | 0.5151666666666667 |
| 8 | 0.10883333333333334 | 0.7360000000000001 |
| 10 | 0.10883333333333334 | 0.7360000000000001 |
| 12 | 0.12649999999999997 | 0.7263333333333334 |
| 20 | 0.3165 | 0.7105 |
| 24 | 0.5221666666666667 | 0.6486666666666667 |(B) N1-227
(F) NRRL B 33157
### Chart
| Category | 0S | p1 |
|---|---|---|
| 0 | 0.11416666666666665 | 0.11183333333333333 |
| 2 | 0.1085 | 0.10683333333333334 |
| 4 | 0.11266666666666665 | 0.11449999999999999 |
| 6 | 0.11266666666666665 | 0.18033333333333335 |
| 8 | 0.10933333333333334 | 0.3358333333333334 |
| 10 | 0.10616666666666667 | 0.5458333333333333 |
| 12 | 0.10483333333333333 | 0.5865 |
| 20 | 0.167 | 0.6153333333333334 |
| 24 | 0.3561666666666667 | 0.5556666666666666 |
### Chart
| Category | 0S | p1 |
|---|---|---|
| 0 | 0.11033333333333334 | 0.13133333333333333 |
| 2 | 0.1035 | 0.11216666666666665 |
| 4 | 0.10683333333333334 | 0.1325 |
| 6 | 0.10466666666666667 | 0.2578333333333333 |
| 8 | 0.1075 | 0.5506666666666666 |
| 10 | 0.12283333333333334 | 0.703 |
| 12 | 0.16783333333333336 | 0.6833333333333332 |
| 20 | 0.26299999999999996 | 0.6181666666666666 |
| 24 | 0.32016666666666665 | 0.6148333333333333 |(C) ScottA
(G) ATCC 43527
### Chart
| Category | 0S | p1 |
|---|---|---|
| 0 | 0.12 | 0.11649999999999999 |
| 2 | 0.11416666666666665 | 0.1105 |
| 4 | 0.1205 | 0.12016666666666666 |
| 6 | 0.17933333333333334 | 0.1815 |
| 8 | 0.32149999999999995 | 0.3223333333333333 |
| 10 | 0.5686666666666668 | 0.539 |
| 12 | 0.6551666666666667 | 0.5325 |
| 20 | 0.7216666666666667 | 0.6821666666666667 |
| 24 | 0.6688333333333333 | 0.6526666666666666 |
### Chart
| Category | 0S | p1 |
|---|---|---|
| 0 | 0.1195 | 0.1195 |
| 2 | 0.11483333333333333 | 0.11549999999999999 |
| 4 | 0.12266666666666666 | 0.13683333333333333 |
| 6 | 0.2038333333333333 | 0.2836666666666667 |
| 8 | 0.39516666666666667 | 0.5991666666666667 |
| 10 | 0.6445 | 0.7395 |
| 12 | 0.6928333333333333 | 0.6986666666666667 |
| 20 | 0.6298333333333334 | 0.6065 |
| 24 | 0.5901666666666666 | 0.5608333333333333 |(D) NRRL B 33109
(H) ATCC 19116
Time (h)
Time (h)
Figure S2. Changes in growth curves (OD600) of QAC-P2 (open squares) in ciprofloxacin-containing broth (2 µg/mL) compared to non-adapted control (open circles) of eight L. monocytogenes strains at 37°C. Error bars indicate standard error of means.
33

## Slide 3
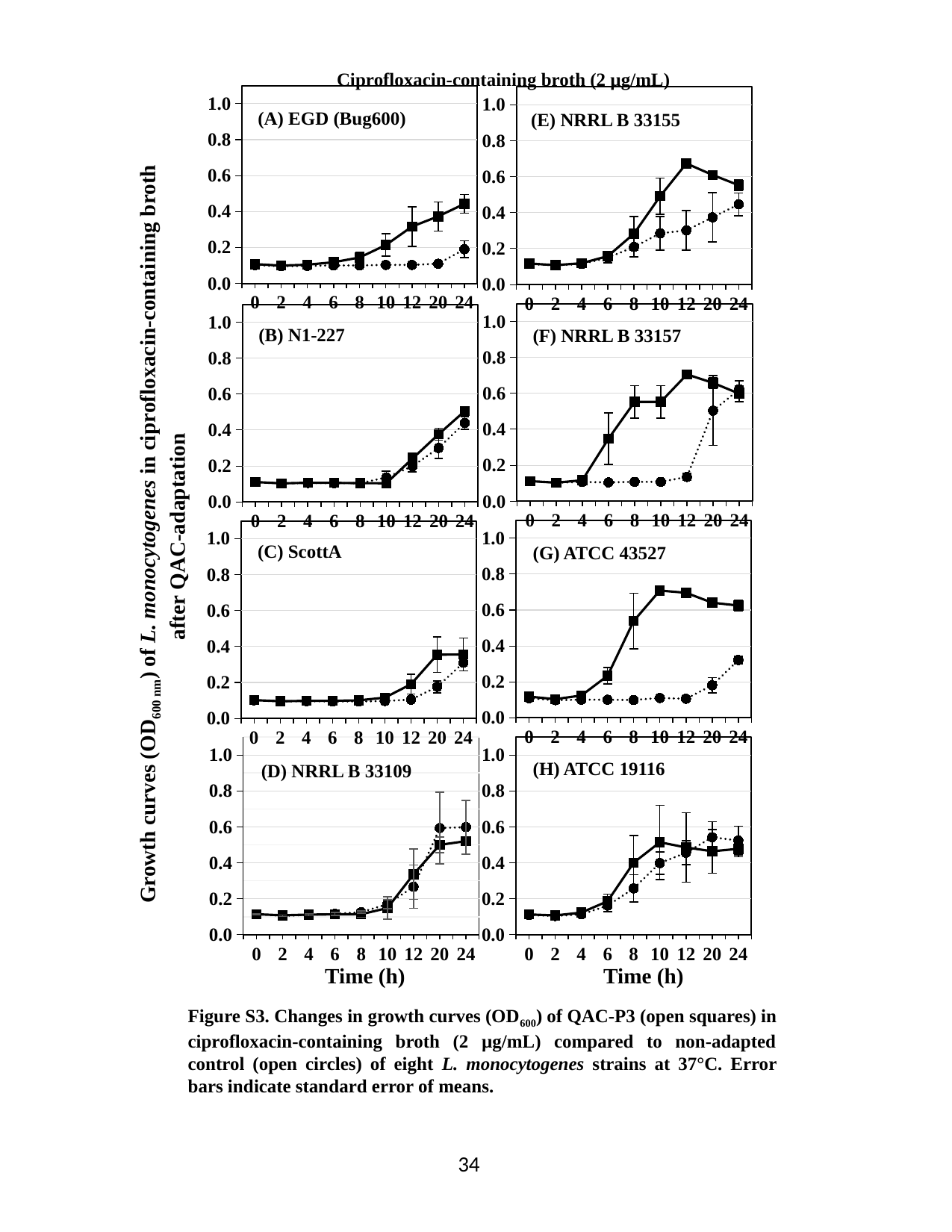

Ciprofloxacin-containing broth (2 µg/mL)
### Chart
| Category | 0S | p1 |
|---|---|---|
| 0 | 0.10166666666666668 | 0.10683333333333334 |
| 2 | 0.09500000000000001 | 0.09866666666666668 |
| 4 | 0.09716666666666667 | 0.10366666666666667 |
| 6 | 0.09949999999999999 | 0.11733333333333333 |
| 8 | 0.09966666666666668 | 0.144 |
| 10 | 0.1025 | 0.21316666666666664 |
| 12 | 0.1025 | 0.31583333333333335 |
| 20 | 0.10933333333333334 | 0.37266666666666665 |
| 24 | 0.18966666666666665 | 0.442 |
### Chart
| Category | 0S | p1 |
|---|---|---|
| 0 | 0.11433333333333333 | 0.11666666666666665 |
| 2 | 0.1065 | 0.10783333333333334 |
| 4 | 0.11416666666666665 | 0.11866666666666666 |
| 6 | 0.14933333333333335 | 0.15816666666666668 |
| 8 | 0.20916666666666664 | 0.2833333333333333 |
| 10 | 0.2846666666666667 | 0.49183333333333334 |
| 12 | 0.30150000000000005 | 0.6738333333333334 |
| 20 | 0.3738333333333334 | 0.6101666666666666 |
| 24 | 0.44616666666666666 | 0.5528333333333334 |(A) EGD (Bug600)
(E) NRRL B 33155
Growth curves (OD600 nm) of L. monocytogenes in ciprofloxacin-containing broth after QAC-adaptation
### Chart
| Category | 0S | p1 |
|---|---|---|
| 0 | 0.112 | 0.11183333333333334 |
| 2 | 0.1035 | 0.10300000000000002 |
| 4 | 0.10683333333333334 | 0.11666666666666665 |
| 6 | 0.10466666666666667 | 0.3481666666666667 |
| 8 | 0.10783333333333334 | 0.5528333333333334 |
| 10 | 0.10783333333333334 | 0.5528333333333334 |
| 12 | 0.135 | 0.7058333333333334 |
| 20 | 0.5041666666666667 | 0.6583333333333333 |
| 24 | 0.6231666666666666 | 0.5985 |
### Chart
| Category | 0S | p1 |
|---|---|---|
| 0 | 0.11033333333333332 | 0.11083333333333334 |
| 2 | 0.10316666666666667 | 0.1035 |
| 4 | 0.10566666666666667 | 0.10683333333333334 |
| 6 | 0.105 | 0.10616666666666667 |
| 8 | 0.10416666666666667 | 0.10466666666666667 |
| 10 | 0.13649999999999998 | 0.10400000000000002 |
| 12 | 0.19866666666666666 | 0.24135000000000004 |
| 20 | 0.30050000000000004 | 0.37670000000000003 |
| 24 | 0.43956666666666666 | 0.5060666666666668 |(B) N1-227
(F) NRRL B 33157
### Chart
| Category | 0S | p1 |
|---|---|---|
| 0 | 0.1085 | 0.11783333333333333 |
| 2 | 0.09783333333333333 | 0.10383333333333333 |
| 4 | 0.10099999999999999 | 0.124 |
| 6 | 0.10058333333333332 | 0.23383333333333334 |
| 8 | 0.09913333333333334 | 0.5386666666666666 |
| 10 | 0.11018333333333334 | 0.7081666666666666 |
| 12 | 0.10586166666666667 | 0.6960000000000001 |
| 20 | 0.18083333333333332 | 0.6406666666666667 |
| 24 | 0.32149999999999995 | 0.6248333333333332 |
### Chart
| Category | 0S | p1 |
|---|---|---|
| 0 | 0.10066666666666667 | 0.10200000000000002 |
| 2 | 0.09483333333333333 | 0.09566666666666668 |
| 4 | 0.09533333333333334 | 0.09783333333333333 |
| 6 | 0.09483333333333333 | 0.09800000000000002 |
| 8 | 0.09400000000000001 | 0.10049999999999999 |
| 10 | 0.09683333333333333 | 0.11566666666666668 |
| 12 | 0.10463333333333334 | 0.19016666666666668 |
| 20 | 0.17600000000000002 | 0.3546666666666667 |
| 24 | 0.3095 | 0.35600000000000004 |(C) ScottA
(G) ATCC 43527
### Chart
| Category | 0S | p1 |
|---|---|---|
| 0 | 0.11399999999999999 | 0.11366666666666665 |
| 2 | 0.10783333333333334 | 0.10516666666666667 |
| 4 | 0.11183333333333333 | 0.10983333333333332 |
| 6 | 0.11316666666666668 | 0.11583333333333334 |
| 8 | 0.11399999999999999 | 0.12516666666666668 |
| 10 | 0.14816666666666667 | 0.16983333333333336 |
| 12 | 0.33616666666666667 | 0.2665 |
| 20 | 0.5 | 0.5933333333333333 |
| 24 | 0.5206666666666667 | 0.5986666666666666 |
### Chart
| Category | 0S | p1 |
|---|---|---|
| 0 | 0.10933333333333334 | 0.11283333333333334 |
| 2 | 0.10416666666666667 | 0.107 |
| 4 | 0.11333333333333333 | 0.123 |
| 6 | 0.16183333333333336 | 0.18616666666666667 |
| 8 | 0.25733333333333336 | 0.4008333333333334 |
| 10 | 0.3985 | 0.5143333333333334 |
| 12 | 0.45583333333333337 | 0.48599999999999993 |
| 20 | 0.5425000000000001 | 0.464 |
| 24 | 0.5241666666666667 | 0.47816666666666663 |(H) ATCC 19116
(D) NRRL B 33109
Time (h)
Time (h)
Figure S3. Changes in growth curves (OD600) of QAC-P3 (open squares) in ciprofloxacin-containing broth (2 µg/mL) compared to non-adapted control (open circles) of eight L. monocytogenes strains at 37°C. Error bars indicate standard error of means.
34
